# High-G Calibration Denoising Method for High-G MEMS Accelerometer Based on EMD and Wavelet Threshold

**DOI:** 10.3390/mi10020134

**Published:** 2019-02-18

**Authors:** Qing Lu, Lixin Pang, Haoqian Huang, Chong Shen, Huiliang Cao, Yunbo Shi, Jun Liu

**Affiliations:** 1 Science and Technology on Electronic Test & Measurement Laboratory, North University of China, Tai Yuan 030051, China; 1606014307@st.nuc.edu.cn (Q.L.); shenchong@nuc.edu.cn (C.S.); shiyunbo@nuc.edu.cn (Y.S.); 2 APT Mobile Satcom Limited, Shenzhen 518126, China; plx@apsatcom.com; 3 College of Energy and Electrical Engineering, Hohai University, Nanjing 210098, China; qhbhhq@163.com

**Keywords:** MEMS accelerometer, noise reduction, EMD, wavelet threshold, Hopkinson Bar, High-G calibration

## Abstract

High-G MEMS accelerometers have been widely used in monitoring natural disasters and other fields. In order to improve the performance of High-G MEMS accelerometers, a denoising method based on the combination of empirical mode decomposition (EMD) and wavelet threshold is proposed. Firstly, EMD decomposition is performed on the output of the main accelerometer to obtain the intrinsic mode function (IMF). Then, the continuous mean square error rule is used to find energy cut-off point, and then the corresponding high frequency IMF component is denoised by wavelet threshold. Finally, the processed high-frequency IMF component is superposed with the low-frequency IMF component, and the reconstructed signal is denoised signal. Experimental results show that this method integrates the advantages of EMD and wavelet threshold and can retain useful signals to the maximum extent. The impact peak and vibration characteristics are 0.003% and 0.135% of the original signal, respectively, and it reduces the noise of the original signal by 96%.

## 1. Introduction

The MEMS accelerometer is a new kind of sensor that is made by microelectronics and micro-machining technology. Compared with the traditional sensor, it has the characteristics of small volume, light weight, low power consumption, high reliability, high sensitivity, easy integration and so on [1,2]. High-G MEMS accelerometers are mainly used for the measurement and control of speed changes of high-speed moving carriers during their start-up and operation. They are widely used in the aerospace field and the precise control of missiles and intelligent projectiles. Therefore, it is important to improve the accuracy of such sensors [3]. As a sensitive source of input systems, High-G MEMS accelerometers are the key to system accuracy. However, due to the influence of the accelerometer itself and signal hardware acquisition circuit, a large number of noise signals will be superimposed on the acquired accelerometer signals, which requires corresponding denoising processing [4,5,6,7,8,9,10]. At present, the widely used Fourier transform and Kalman Filter [11] have many defects. The Fourier Transform needs to extract the signal with all the information in the time domain, which is a kind of integral transformation. It lacks the function of time domain positioning, and the resolution is lower when applied in the time domain. Moreover, the Fourier Transform does not reflect the case where the instantaneous frequency of the signal changes over time. It is more suitable for analyzing the stationary signal. For the non-stationary signal whose frequency changes with time, the Fourier Transform can only give its overall effect and cannot fully grasp the essential characteristics of the signal at a certain moment. However, Short-term Fourier Transform can be used for non-stationary signals, and in fact is common in speech processing. The matrix operation used in the denoising of Kalman Filter makes the calculation time longer and the waveform distortion more serious. In recent years, many achievements have been made in the study of signal denoising method based on EMD. Empirical mode decomposition (EMD) is a new method for nonlinear and non-stationary data analysis proposed by Dr. Norden Huang. It allows any complex data set to be decomposed into a finite number of intrinsic mode functions (IMF). Since the decomposition method is based on local characteristics of data time scale, it can be applied to the processing of non-linear and non-stationary signals [12,13]. Wang et al. [14,15,16] treated the functional components of each mode obtained by EMD decomposition with threshold value, and then reconstructed them to achieve the purpose of noise removal. However, they ignored that the high-frequency noise mode function components of low order cannot be completely eliminated through the threshold processing. Boudraa et al. [17] determined the demarcation point between signal area and noise area based on energy criteria, but there was a defect of poor stability. Chen et al. [18] determined the demarcation point according to the correlation coefficient criterion between the intrinsic mode function of each order and the original signal, and obtained good analysis results, but ignored the noise of higher-order mode function components. As a relatively mature signal analysis method, wavelet transform has been widely used in signal de-noising due to its multi-scale, de-correlation and low entropy properties, especially in the suppression of random noises. Xu et al. [19] proposed a threshold denoising method based on wavelet analysis, which can obtain the best estimation value in Besov space. Bi et al. [20] combine EMD and wavelet transform for engine blasting feature detection. This method is suitable for engine knock signals with non-stationary and transient characteristics and can identify the tapping characteristics of vibration signals. More reliable, faster and more efficient than previous signal processing methods. In this paper, EMD decomposition is combined with wavelet threshold denoising method, and continuous mean square error criterion is introduced to obtain energy demarcation points to determine the high-frequency IMF component that needs noise reduction. Only the high-frequency IMF component is denoised by wavelet threshold, and the low-frequency IMF component remains unchanged. The signal after noise reduction is obtained through signal reconstruction. This is a new method of noise reduction, which avoids the disadvantage of direct loss of useful information on high frequency components caused by EMD. Moreover, such wavelet threshold denoising only acts on the high frequency IMF component, rather than on the entire signal, largely overcoming the shortcomings of the direct wavelet threshold denoising method. It is much more accurate than using EMD and wavelet thresholds singly. We use the continuous mean square error criterion to determine the energy boundary point. The continuous mean square error criterion is more accurate, stable and reliable than other boundary methods such as artificially independent boundary, correlation coefficient criterion and energy criterion. This joint denoising method is suitable for the removal of high overload MEMS accelerometer signals with shock and vibration characteristics. Experimental results and discriminant results can verify the advantages of the combined denoising method proposed in this paper. The impact peak and vibration characteristics are 0.003% and 0.135% of the original signal, respectively, and it reduces the noise of the original signal by 96%. In order to improve the performance of MEMS accelerometer during High-G calibration, a new combined desensitization method based on EMD and wavelet threshold is proposed. The structure of this paper is as follows: part 2 describes the proposed algorithm, part 3 introduces the accelerometer, part 4 gives the experiment and verification, and the last part is the conclusion.

## 2. Algorithm

### 2.1. Empirical Mode Decomposition (EMD)

Empirical mode decomposition is to decompose the signal into several intrinsic mode functions and a residual component. The first step of the decomposition process [21] is to find all the maximum points of the original data sequence *x*(*t*) and fit them using a cubic spline interpolation function to form the upper envelope of the original data. Similarly, all the minimum points are found, and all the minimum points are fitted by the cubic spline interpolation function to form the lower envelope of the data. The average value of upper envelope line and lower envelope line is denoted as *m*_1_(*t*), and the original data sequence *x*(*t*) is subtracted from this average envelope *m*_1_(*t*) to obtain new data sequence *h*_1_(*t*), that’s:

(1) $$x(t)-m_{1}(t)=h_{1}(t)$$

If *h*_1_(*t*) is not an intrinsic mode function, then smoothing is required. In the second smoothing process, *h*_1_(*t*) is the original data, that is:

(2) $$h_{1}(t)-m_{11}(t)=h_{11}(t)$$

where *m*_11_(*t*) is the average of the upper and lower envelope of *h*_1_(*t*), repeat the above smoothing process *k* times until *h*_1*k*_(*t*) is an intrinsic mode function, that is:

(3) $$h_{1(k-1)}(t)-m_{1k}(t)=h_{1k}(t)$$

Then, *h*_1*k*_(*t*) is the first-order IMF classification, denoted by *a*_1_, and *a*_1_ is separated from the original sequence to obtain the residual term *r*_1_:

(4) $$r_{1}=x(t)-a_{1}$$

After that, we use the residual item *r*_1_ as the new data, and smooth it with the same method as before to get the new residual item *r*_2_ = *r*_1_ − *a*_1_. The above process is repeated until *r_n_* = *r_n_*_−1_ − *a_n_* is less than the given value, or the residual term becomes a monotonic function or has at most one maximum point, that is, it is impossible to extract an IMF component from it, and then the model decomposition process is terminated. Finally, the original signal *x*(*t*) can be composed of *n* IMF components and residual *r_n_*:

(5) $$x(t)=\sum_{i=1}^{n}a_{i}+r_{n}$$

### 2.2. Wavelet Threshold Denoising

Signal de-noising is essentially a process of suppressing the useless part of the signal and enhancing the useful part of the signal. Generally, the process of wavelet threshold denoising can be divided into the following three steps [22,23]: 

(1) Wavelet decomposition. Select a wavelet and determine the level of decomposition, then the decomposition calculation.

(2) Threshold quantization of wavelet decomposition high frequency coefficient. A threshold value is selected for the high frequency coefficients of each decomposition scale to be quantized, and the low frequency wavelet coefficient is kept unchanged.

(3) Wavelet reconstruction. The processed high frequency wavelet coefficients and low frequency wavelet coefficients are reconstructed by inverse wavelet transformation, and the signal after noise reduction is obtained. 

The wavelet threshold denoising method adopts different thresholds based on different scales, that is, with the increase of scales, the threshold value gradually decreases, making the propagation characteristics of noise in different scales of wavelet transform consistent. This method is also applicable to the removal of relevant noise. In addition, the threshold can be changed with the change of the position of the coefficient on the same scale, that is, the adaptive threshold denoising in the wavelet domain. The wavelet threshold denoising algorithm needs to determine a threshold value. The effect of wavelet threshold denoising [24] is closely related to the selection of threshold estimation and threshold function, and there are a variety of determination criteria. Common threshold selection methods include fixed threshold estimation (sqtwolog), maximal minimum threshold estimation (minimaxi), unbiased risk estimation (rigsure), heuristic threshold estimation (heursure), etc. In this paper, unbiased likelihood estimation (rigsure) suitable for high frequency components is selected. The wavelet coefficients larger than this threshold value are considered to be generated by the effective signal, while the wavelet coefficients smaller than this threshold value are considered to be generated by noise. The algorithm is:

(1) The elements in the vector to be estimated are taken as absolute values, then sorted from small to large, and then each element is squared to obtain a new vector *NV* to be estimated whose length *n* is the length of the original estimated vector.

(2) Corresponding to the subscript *k* of each element, if the threshold is the square root of the *k*-th element of the vector to be estimated, the risk algorithm is:

(6) $$Risk(k)=\frac{n-2k+\sum_{j=1}^{k}NV(j)+(n-k)NV(n-k)}{n}$$

(3) According to the above formula, the minimum risk point and the corresponding *k* value are found, and the threshold is:

(7) $$Thr=\sqrt{NV(k)}$$

The threshold processing method includes a hard threshold and a soft threshold method. The hard threshold method keeps the wavelet coefficients higher than the threshold unchanged and sets the wavelet coefficients of each subspace below the threshold to zero; the soft threshold method is to apply the wavelet coefficients to a fixed amount. Shrink to zero, reconstructed by new wavelet coefficients to obtain denoised signals.

The hard threshold value is expressed as:

(8) $$\eta \left(a,b\right)=\{\begin{array}{ll} 0 & \left|a\right|<b\\ \left|a\right| & \left|a\right|\geq b \end{array}$$

Soft threshold is expressed as:

(9) $$\eta \left(a,b\right)=\{\begin{array}{ll} 0 & \left|a\right|<b\\ sign\left(a\right)\left(\left|a\right|-b\right) & \left|a\right|\geq b \end{array}$$

In the above two equations, *a* is the wavelet coefficient and *b* is the threshold. $b=\sigma \times \sqrt{2\mathrm{lg}N}$, where σ is the noise standard value and *N* is the signal length. Noise standard value $\sigma =\frac{Median\left(\left|a\right|\right)}{0.6745}$, of which Median(|a|) is the median of wavelet multi-resolution decomposition coefficient [25].

### 2.3. Wavelet Thresholding Denoising Based on EMD

For the signals with small amplitude of the useful signal, which are overwhelmed by noise signal to a large extent, the effect of using wavelet analysis to remove noise is not very good at this time. Moreover, the EMD-based spatial-temporal filtering algorithm simply removes one or more IMF components to achieve filtering, resulting in the deletion of useful signals on corresponding components together. Therefore, it is a very rough denoising method, which will lead to serious signal distortion. In this paper, EMD and wavelet threshold denoising are combined. First, the original signal is decomposed by EMD to obtain IMF components with frequency ranging from high to low. Then, only high-frequency IMF components are denoised by wavelet threshold, while low-frequency IMF components remain unchanged. Finally, the denoised signal is obtained by combining the denoised high frequency IMF component with the untreated low frequency IMF component and the residual amount. In order to define which IMF components need to be denoised by wavelet threshold and which do not need to be processed, the cut-off point is determined by the continuous mean square error criterion. Since IMF component satisfies the rule of distribution from high frequency to low frequency, while IMF component of low frequency band is dominated by signal, noise mainly exists in IMF of high frequency band. Therefore, there must be some component *A_ks_*, dividing IMF component into two sets of high frequency band and low frequency band. This component can be calculated explicitly and uniquely by the algorithm, which further improves the accuracy of the denoised result compared to other methods. Define the reconstructed signal ${\hat{x}}_{i}\left(t\right)$ as:

(10) $${\hat{x}}_{i}\left(t\right)=\sum_{k=i}^{n}A_{k}\left(t\right)+r_{n}\left(t\right)$$

where, *A_k_*(*t*) is the *k*-th IMF component of signal *x*(*t*) obtained by EMD. The continuous root mean square error of the signal is defined as:

(11) $$\begin{array}{ll} \sigma_{CMSE}\left({\hat{x}}_{i},{\hat{x}}_{i+1}\right) & =\frac{1}{N}\sum_{i=1}^{N}{\left[{\hat{x}}_{i}\left(t\right)-{\hat{x}}_{i+1}\left(t\right)\right]}^{2}\\ & =\frac{1}{N}\sum_{i=1}^{N}{\left[A_{i}\left(t\right)\right]}^{2} \end{array}$$

where *N* is the length of signal *x*(*t*), and *A_i_*(*t*) is the *i*-th IMF component of signal *x*(*t*) obtained by EMD decomposition. Based on the above analysis, it can be determined that the signal energy demarcation point is:

(12) $$k_{s}=\underset{1\leq i\leq n-1}{\arg\min}\left[\sigma_{CMSE}\left({\hat{x}}_{i},{\hat{x}}_{i+1}\right)\right]$$

After the cut-off point, *A_ks_* is determined, the IMF component is divided into two parts. In the first part, IMF components in high frequency band are denoised by wavelet threshold, while in the second part, IMF components in low frequency band are not processed. In this way, it can overcome the shortcoming of the simple EMD denoising method in suppressing the effective information of high frequency while removing the high frequency noise. Based on the above analysis, the specific steps of the de-noising algorithm proposed in this paper are as follows:

(1) EMD decomposition is carried out on the original noise signal *x*(*t*), and each modal component *A_k_* is obtained;

(2) According to the continuous mean square error criterion, the value of *k_s_* is determined.

(3) The threshold determination criteria were selected to calculate the threshold of each mode component *A*_1_–*A_k_*, and wavelet threshold denoising was performed on the modal component *A*_1_–*A_k_*, to obtain each mode component *Â*_1_*–Â_k_* after denoising.

(4) Reconstructed signal, $\bar{x}\left(t\right)=\sum_{k=1}^{k_{s}}{\bar{A}}_{k}\left(t\right)+\sum_{k=k_{s}+1}^{n-1}A_{k}\left(t\right)+r_{n}\left(t\right)$.

**Figure 1:**
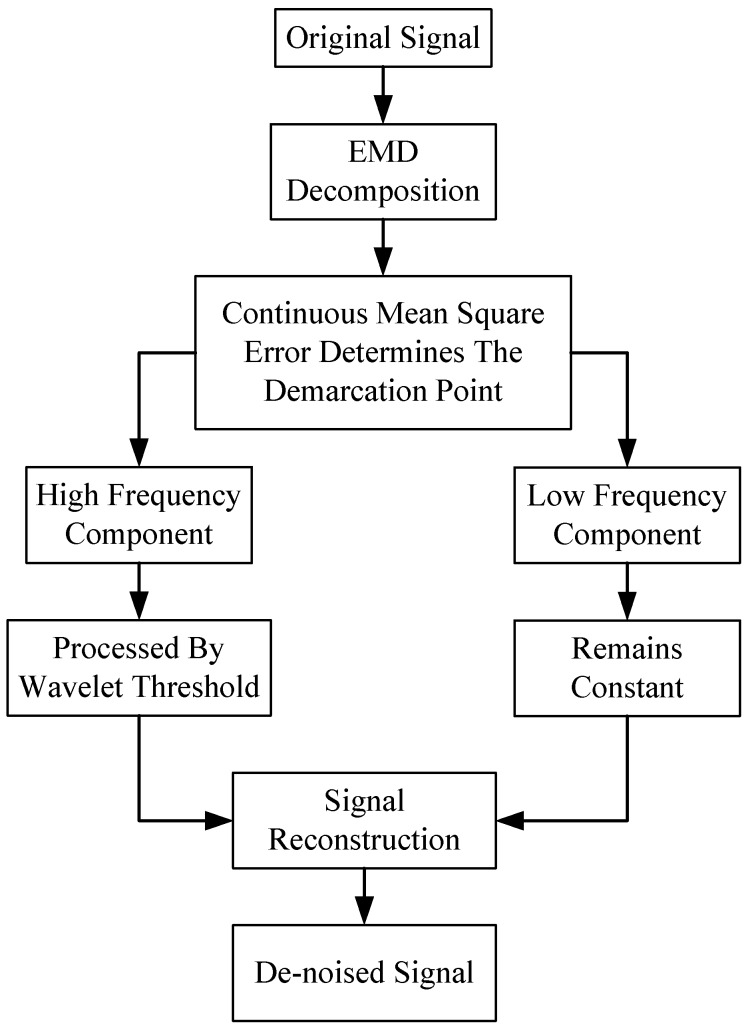
Block diagram based on empirical mode decomposition (EMD) wavelet threshold algorithm.

The algorithm flow chart is shown in Figure 1:

## 3. High-G MEMS Accelerometer

The original signal collected in this paper was from a newly designed and manufactured high-G MEMS accelerometer (HGMA) [26,27]. It is a kind of accelerometer with high impact survival rate and high range. The detection method of the HGMA is piezoelectric resistance, and output signal is voltage. 

### Structure and Structural Parameters of the HGMA

**Figure 2:**
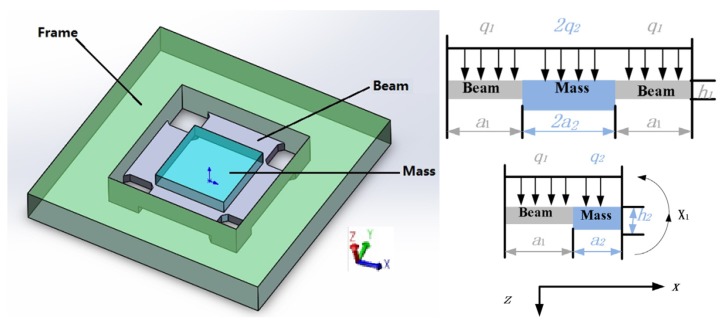
High-G MEMS accelerometer (HGMA) structure schematic and size.

HGMA adopts four beams and island structure [27]. The frame, four beams and the center mass are all rectangular, which is conducive to processing. Its structure diagram and parameters are shown in Figure 2. 

**Figure 3:**
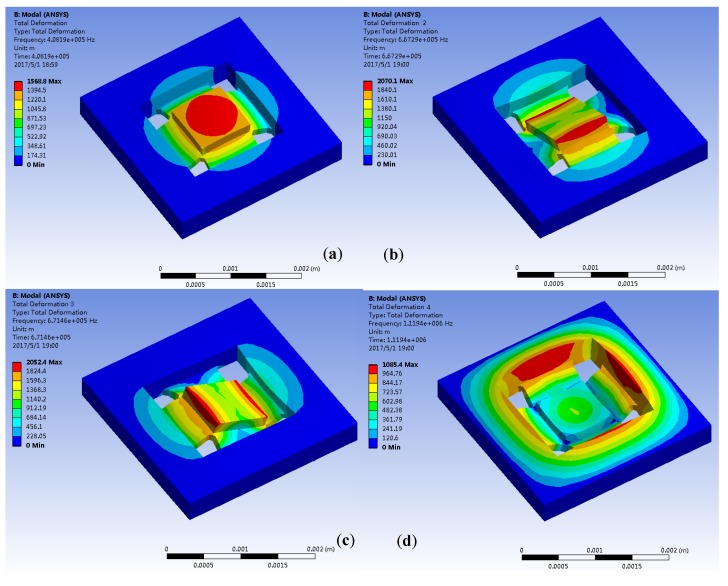
Mode simulation of HGMA structure (**a**–**d**) are 1st, 2nd, 3rd and 4th order modes.

The coordinate system is constructed with the cross-section of the accelerometer. The central dividing line of the cross-section is *Z* axis, specify that the direction is positive to the downward. The other middle line is the *X* axis, and the right direction is positive. The frame constructed is shown in Figure 3. The beam’s length, width and thickness are *a*_1_, *b*_1_ and *c*_1_, respectively. The mass’s length, width and thickness are *a*_2_, *b*_2_ and *c*_2_, respectively. The size values are shown in Table 1.

The first four orders are simulated and analyzed through ANSYS software (ANSYS, Inc., Southpointe, PA, USA) and are shown in Figure 3a–d are the first, second, third and fourth modes respectively. The first mode mass moves along the *Z* axis and is the working mode; The second mode mass rotates around the *X*-axis; The third mode mass rotates around the *Y*-axis; The fourth mode mass and frame move along the *Z* axis. The resonant frequencies of the four modes are shown in Table 2, which indicates that the 1st order is the working mode of HGMA and its resonant frequency is 408 kHz. The 2nd order mode resonant frequency is 667 kHz and has 260 kHz gap with 1st mode, which means the coupling movement between these two modes is tiny and is good for HGMA linearity. 

**Figure 4:**
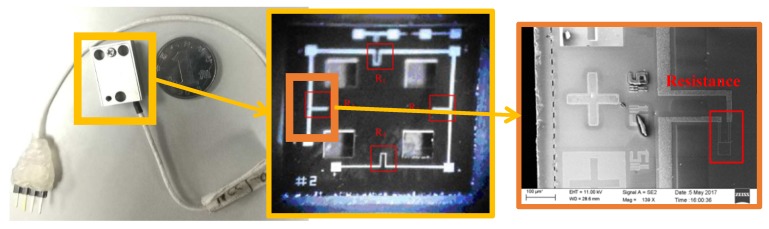
Overall photo, confocal microscopy photo and SEM photo of HGMA.

The structure of HGMA is made of silicon and bonding on glass, and the main technological process is mainly divided into 12 steps. And the SEM photos and confocal microscopy photos of the accelerometer structure are shown in Figure 4 [27].

## 4. Experiment and Verification

### 4.1. Experiment

**Figure 5:**
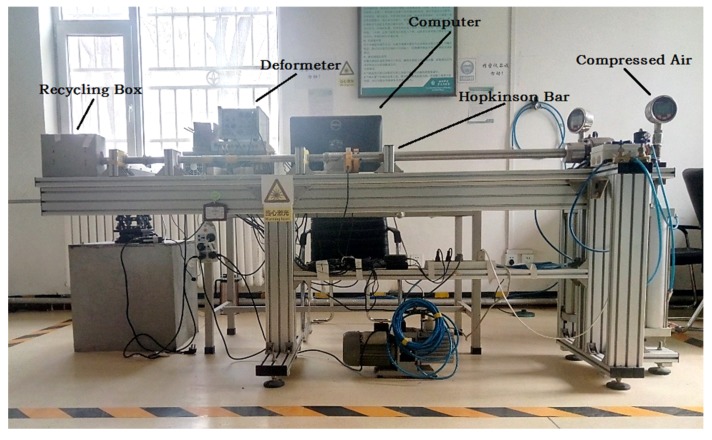
Hopkinson Bar calibration system.

The HGMA was calibrated in Hopkinson Bar calibration system as shown in Figure 5, and its output signal was also collected, and the equipment is shown in Figure 5. A power supply was employed to provide +5 V voltage to HGMA, and a high-speed data acquisition system and a computer were employed to collect the HGMA output signal. The temperature was 25 °C (room temperature value), and the sampling rate was 20 MHz.

### 4.2. Verification

(1) EMD Denoising

**Figure 6:**
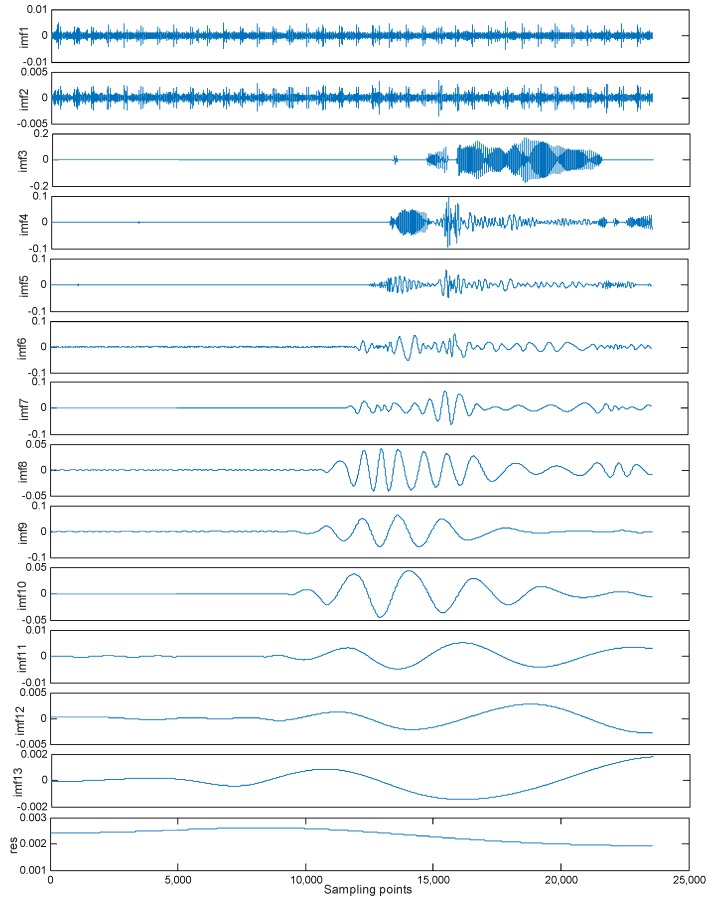
EMD decomposition of the original signal.

**Figure 7:**
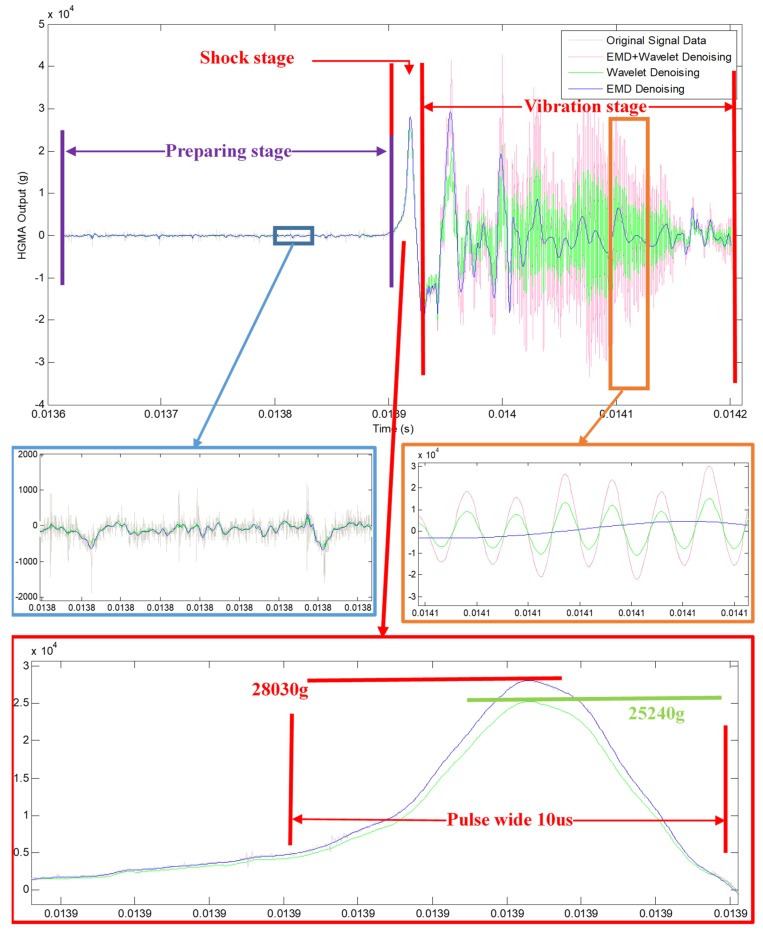
Signals before and after denoising.

EMD decomposition was performed on the original signal data. The original signal data was decomposed into IMF1–IMF13, a total of 13 components and 1 residual component res. The exploded view is shown in Figure 6. It can be seen from the figure that noise mainly existed in the first three components. According to the principle of EMD low-pass filtering de-noising method, the first three high-frequency components were removed, and the remaining components were reconstituted as signals. The reconstructed signal is shown in Figure 7. It can be seen from the figure that the signal after denoising is clear and smooth, the noise is effectively removed, but the signal amplitude is significantly reduced. This is because the first three components that were discarded contain both noise and useful information. The direct EMD denoising method can reduce useful signals while removing high frequency noises, resulting in information loss. Therefore, this method is relatively rough.

(2) Wavelet Threshold Denoising

After comparing the effects of several wavelet functions and decomposition layers, finally chose the ‘db4’ wavelet as the wavelet generating function and set the decomposition scale to 4. And the corresponding soft threshold was applied to the high frequency coefficient. Figure 7 shows the result of wavelet denoising directly to the original signal. It can be seen from Figure 7 that the wavelet threshold denoising method can directly remove the noise of the high frequency part and restore the original signal, and the decimation effect of the low frequency part is much better than the EMD. The denoising effect is relatively ideal, but the signal amplitude after denoising is still reduced, indicating that there are still useful signals removed.

(3) Wavelet Threshold Denoising based on EMD

Since the EMD denoising method discards one or more high-frequency components, the high-frequency noise was removed, and the effective information on the corresponding components was also removed together, thereby causing severe distortion of the signal. The wavelet threshold denoising method removes most of the noise and removed the small effective signal together, which also caused certain errors. Therefore, the combination of EMD and wavelet threshold was used for denoising. According to the continuous mean square error criterion, the cut-off point was IMF3. Therefore, the first three components after EMD decomposition were processed by wavelet threshold, while the rest components remained unchanged. The processed component was reconstructed with other unprocessed components, and the result after denoising was obtained. The reconstruction signal is shown in Figure 7. As can be seen from Figure 7, the noise is effectively removed, and the signal after joint denoising is relatively smooth, substantially free of burrs, the amplitude is substantially unchanged, and useful information is retained. The denoising effect is ideal.

The original signal data and denoising results are shown in Figure 7, it can be seen from the original signal data that three main stages are divided:

1. **Preparing stage**: before the shock peak, and this part contains the noise signal and the bias characteristic of HGMA. As can be seen from Figure 7, the noise of original signal data is large (peak-peak value is near about 1000 g), and EMD, Wavelet and EMD + Wavelet methods all work well, and which are proved by denoising results.
2. **Shock stage**: the main part of the calibration experiment, the peak value is about 28,030 g, and the pulse wide is about 10 μs. During this stage, the original signal data, EMD and EMD + Wavelet denoising signals almost overlapping, which indicates that these three curves contain the same information. However, the Wavelet denoising signal amplitude is 25,240 g, which is not the real peak value of original signal data, and the error is more than 10%. So, Wavelet method is not suitable for the calibration denoising.
3. **Vibration stage**: after the shock peak, and this part mainly contains HGMA vibration information, which reflects the dynamic characteristic of HGMA. In this stage, it can be seen that the EMD denoising signal occurs distortion phenomenon and cannot reflect the frequency and amplitude information of original data any more. Meanwhile, the amplitude information of original signal data cannot be expressed after Wavelet denoising. Only EMD + Wavelet method follows the original signal data.

**Figure 8:**
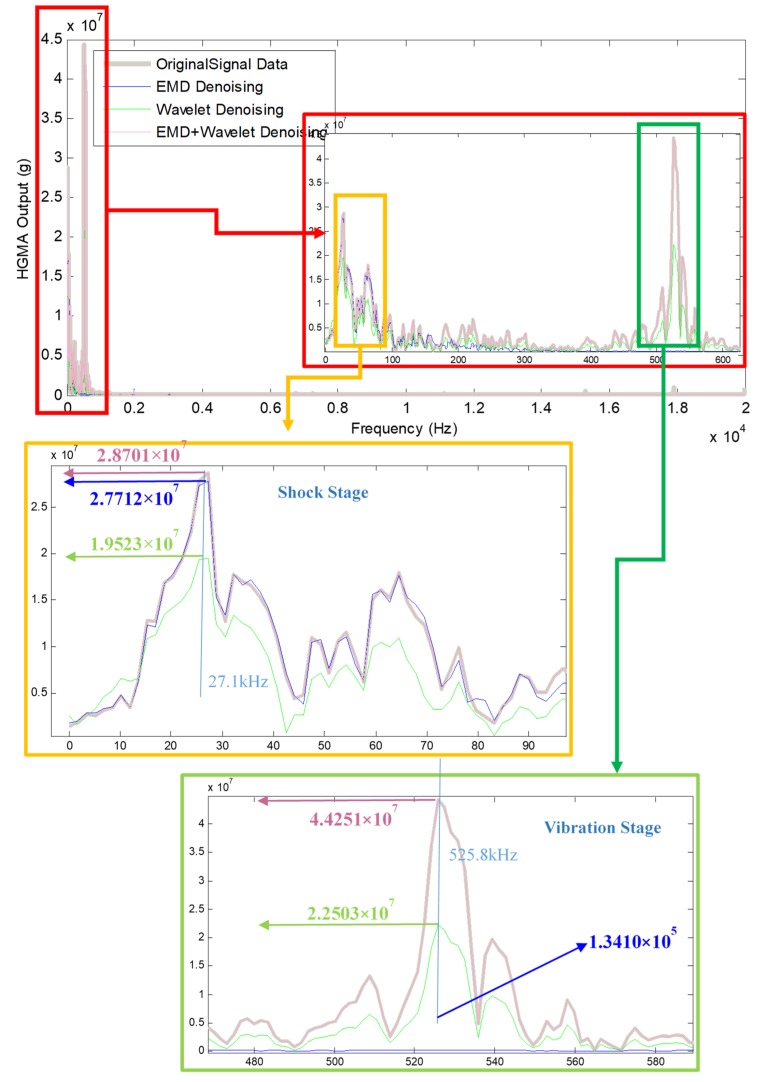
Frequency characteristic of original signal and denoising results.

The frequency characteristic of original signal data and denoising results are shown in Figure 8, the “Shock Stage” and “Vibration Stage” are enlarged:

1. **Shock stage**: the frequency peak of this stage is about 27.1 kHz, the original signal data and EMD + Wavelet denoising results have almost the same amplitude and shape (one amplitude is 2.8702 × 10^7^, the other is 2.8701 × 10^7^); the EMD denoising result amplitude is 2.7712 × 10^7^; the Wavelet denoising result amplitude is 1.9523 × 10^7^, which shows that EMD + Wavelet denoising method inherit the real amplitude and frequency information of original signal.
2. **Vibration stage**: the frequency peak of vibration stage is about 525.8 kHz, the original signal data and EMD + Wavelet denoising results have almost the same amplitude and shape (one amplitude is 4.4310 × 10^7^, the other is 4.4251 × 10^7^); the EMD denoising result amplitude is 1.3410 × 10^5^; the Wavelet denoising result amplitude is 2.2503 × 10^7^, which shows that EMD + Wavelet denoising method inherit the real amplitude and frequency information of original signal.

**Figure 9:**
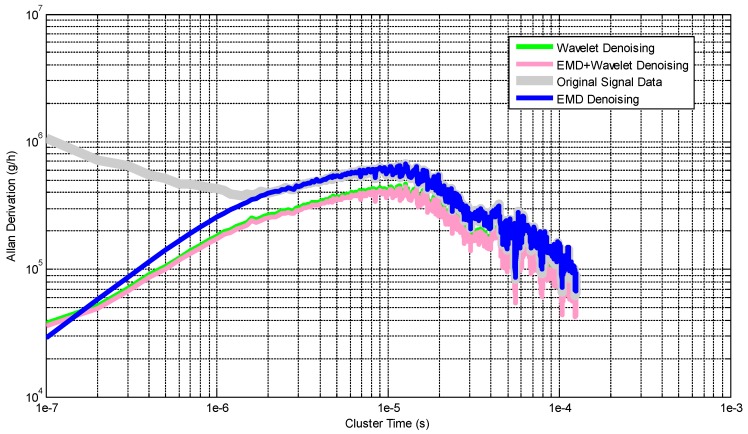
Allan derivation results of the denoising method during “Preparation Stage”.

The Preparing Stage signals are analyzed by Allan Derivation (shown in Figure 9), which is widely used in gyroscope experiment, and the curves can quantitate the equivalent value of the acceleration random walking (which can express the noise characteristic of HGMA). The value of original signal, EMD, Wavelet and EMD + Wavelet denoising signals in 10^−7^ s are 1.0591 × 10^6^ g/h, 2.9241 × 10^4^ g/h, 3.6162 × 10^4^ g/h and 3.7970 × 10^4^ g/h respectively.

Table 3 lists the denoising results of the three methods, as can be seen from Table 3, compared with the wavelet threshold denoising method and the traditional EMD decomposition denoising method, the combined (EMD + Wavelet) denoising method is more effective in “Shock Stage” and “Vibration Stage”. The EMD + Wavelet denoising method errors in these two stages are 0.003% and 0.135% respectively, which shows that the method does not destroy the original calibration data, and can be employed during the calibration process data processing. Meanwhile, the denoising results (the data is picked up in “Preparing Stage”) are also shown in Table 3, the results indicate that three denoising methods achieve excellent results, and they cut more than 96% noise in original signal.

## 5. Conclusions

In this paper, an EMD-based wavelet threshold denoising method was adopted. The cut-off point was determined by the continuous mean square error criterion. Only the IMF components of the previous high frequency bands were selected for wavelet threshold denoising, and no processing was done on the low frequency part. Experiments showed that this method can not only remove part of high frequency noise (96.4% noise component in original signal data), but also retain the details of low frequency (such as shock peak amplitude error is 0.003% and vibration characteristic amplitude error is 0.135% from original signal data). The root mean square error and signal-to-noise ratio are much better than the single method, which can improve the performance of High-G MEMS accelerometer and make it more widely used.

## Figures and Tables

**Table 1:** Structural parameters of the HGMA.

	Beam			Mass		
Parameters	Length (*a*_1_)	Width (*b*_1_)	Height (*c*_1_)	Length (*a*_2_)	Width (*b*_2_)	Height (*c*_1_)
**Size (μm)**	350	800	80	800	800	200

**Table 2:** Resonant frequencies of the four modes.

Mode Shapes	1	2	3	4
**Resonant Frequency (kHz)**	408	667	671	1119

**Table 3:** Comparison of three denoising algorithms.

		EMD	Wavelet	EMD + Wavelet	Original
**Preparing Stage**	**Bias Stability Value @10^−7^s (g/h)**	2.9241 × 10^4^	3.7970 × 10^4^	3.6162 × 10^4^	1.0591 × 10^6^
	**Improves from Original**	97.2%	96.6%	96.4%	-
**Shock Stage**	**Value**	2.7712 × 10^7^	1.9523 × 10^7^	2.8701 × 10^7^	2.8702 × 10^7^
	**Error from Original**	3.49%	32.1%	0.003%	-
**Vibration Stage**	**Value**	1.3410 × 10^5^	2.2503 × 10^7^	4.4251 × 10^7^	4.4310 × 10^7^
	**Error from Original**	99.70%	49.22%	0.135%	-
